# Detection of acrylamide traces in some commonly consumed heat-treated carbohydrate-rich foods by GC-MS/MS in Bangladesh

**DOI:** 10.1016/j.heliyon.2022.e11092

**Published:** 2022-10-13

**Authors:** G. M. M. Anwarul Hasan, Anuj Kumer Das, Mohammed A. Satter

**Affiliations:** a Institute of Food Science and Technology (IFST), Bangladesh Council of Scientific and Industrial Research (BCSIR), Dr. Qudrat-E-Khuda Road, Dhaka 1205, Bangladesh; b Hi-Tech Health Care Ltd. Banani, Dhaka, 1213, Bangladesh

**Keywords:** Acrylamide, Carbohydrate-rich foods, GC-MS/MS, Bangladesh

## Abstract

The present study aimed to determine acrylamide traces in180 heat-treated carbohydrate-rich foods through gas chromatography coupled to mass spectrometry (GC-MS/MS) in Bangladesh. Detected acrylamide contents were 730 ± 293 μg/kg, 244 ± 83 μg μg/kg, 598 ± 222 μg/kg, 340 ± 189 μg/kg, 548 ± 278 μg/kg, 217 ± 77 μg/kg, 558 ± 297 μg/kg, 391 ± 263 μg/kg and 679 ± 285 μg/kg in potato chips, chanachur (a locally processed food), potato crisps, biscuits, cake, bread, crackers, breakfast cereals and French fries respectively. The use of different ingredients during the manufacturing process might affect on acrylamide formation as different ingredients contained variable amounts of free asparagines and reducing sugars to form acrylamide. Among the analyzed samples, 20% of potato chips, 5% of chanachur, 15% of potato crisps, 15% of biscuit, 10% of cake, 15% of bread, 20% of crackers, 10% of breakfast cereal and 20% of French fries samples, representing only a few samples in each category, were found to have acrylamide contents above benchmark levels set by the European Commission [EC]. This study provided an estimation of the presence of acrylamide traces in heat treated carbohydrate rich foods consumed by local population.

## 1 Introduction

Acrylamide is a small unsaturated amide molecule that is absorbed by both humans and animals through food consumption and distributed throughout the body [1, 2]. During processing or cooking at high temperatures, acrylamide is produced in certain foodstuffs, particularly in carbohydrate-rich food [3, 4]. The molecular weight, melting point, vapor pressure and boiling point of acrylamide are 71.08 g/mol, 84.5 ± 0.3 °C, 0.005 mmHg at 25 °C and 136 °C at 3.3 kPa/25 mm Hg, respectively [5]. Acrylamide is a highly water soluble compound [6].

The International Agency for Research on Cancer (IARC) has classified acrylamide as potential carcinogen and after confirmation in animal studies; it has become a significant public health concern [8, 9, 10]. The largest concentrations of acrylamide are found in processed foods such as French fries and chips made from potato [12]. Acrylamide is formed during the browning process when reducing sugars interact with asparagine at high temperature (>120 °C). Sugars and asparagine are the main reactants that cause acrylamide to be produced [3, 4, 13, 14, 15]. Minerals, carbohydrates, proteins, vitamins, lipids, enzymes and other essential nutrients are important food components. During food processing, these food components may lose sensitivity due to light, heat, pH, oxygen or combined effect of these factors [7,11].

In Bangladesh, potato chips, chanachur, potato crisps, biscuits, cakes, and crackers are regularly consumed as snacks. Potato chips are thin potato slices that have been dehydrated to a moisture level of 0.02 kg/kg or less by deep fat frying [16, 17]. Potato chips have a high oil content, ranging from 35 to 45% (wet basis), which gives them a distinctive texture–flavor combination that makes them highly appealing [18, 19]. High drying rates are crucial for achieving desirable structural and textural qualities of the final product during dehydration in hot oil at temperatures between 160 °C and 180 °C [17]. Chanachur is a very common food in the Indian subcontinent and is made of chickpea flour, peanuts, green beans, lentils, split chickpeas, rice flakes, edible oil, spices, salt, citric acid, red chili powder, powder and turmeric powder. During processing, the mix of ingredients is fried with oil. Potato crisps are thin fried slices made from potato paste. The main ingredients of both crackers and biscuits are flour, and other ingredients, such as salts, sugar, flavors and sometimes cheese, are added before baking. Cakes are celebratory food items that are made from flour, eggs, oil, sugar and other ingredients. There are many cake varieties in Bangladesh, and people prefer cakes during celebrations. The main ingredients of bread dough are flour and water. Baking is the final step of cake and bread preparation. Breakfast cereals are prepared from processed cereal grains. Oat meal and corn flakes are commonly consumed breakfast cereals in Bangladesh. People prefer to take these processed foods almost every day either as light meals or as supplementary food with tea and coffee both at home and in the workplace. Bangladeshi people like to have breads or breakfast cereals as their breakfast. French fries are consumed as supplementary fast food items at home and in restaurants.

Acrylamide is generated during food processing. High-temperature-treated processed foods contain high acrylamide concentrations, where a reaction occurs between reducing sugars and asparagines under low moisture conditions [3, 4, 20, 21]. Because of its toxicity, accurate detection of acrylamide in food has become a critical concern in food safety [22]. FAO/WHO on Food Additives (JECFA) joint forum stated about the acrylamide toxicity in food [23].

Acrylamide is analyzed normally in foods through two methods: GC-MS/MS and HPLC–MS/MS. Because of the high polarity, low volatility and low molecular weight of acrylamide, a derivatization process is necessary for acrylamide detection though GC-MS/MS.

As an agricultural country, Bangladesh produces many carbohydrate-rich cereal crops, such as rice, wheat, maize, barley and sorghum. Bangladesh also produces roots and tubers that contain high water (approximately 70–80%) along with high starch contents (approximately 16–24%). Bangladesh is in the 7th position in terms of potato production among countries of the world. The total potato production was 8.6 million tons in Bangladesh, with a consumption of 6.5 million tons in 2014 [24], which indicates that large amounts of potato and processed potato foods such as potato crackers, potato chips, and French fries were consumed in Bangladesh. In tuber crops such as potato, the protein, mineral and vitamin contents are lower than those in cereal crops. Most of the food items of Bangladesh are mainly produced from cereals and tubers either through cooking or thermal processing. As acrylamide is formed in processed carbohydrate-rich foods, it is of great importance to determine the acrylamide content in commonly consumed processed foods in Bangladesh to address public health safety concerns. The aim of the current research was to detect acrylamide contents in heat-treated carbohydrate-rich foodstuffs of Bangladesh by using GC-MS/MS.

## 2 Materials and methods

### 2.1 Chemicals

Chemicals such as Acrylamide with 99% purity, isotope-labeled internal standard (IS) d3, methanol with 99% purity (analytical grade), 99% pure acetonitrile and 99% pure n-hexane were purchased from Sigma–Aldrich (St. Louis, USA) while 99% Magnesium sulfate and 99% pure sodium hyposulfite were brought from Merck (Germany). Analytical grade water was used throughout the analytical process.

### 2.2 Samples collection

Heat treated carbohydrate-rich foods were collected for this analysis. The only criterion followed during sample collection was that the samples were produced from high carbohydrate-rich raw materials and processed through thermal treatment. Foods in those categories, including commercial potato chips, chanachur, bread, biscuits, cake, potato crisps, crackers and breakfast cereals were collected from local shops. We also collected some non-branded potato chips, Chanachur, potato crisps and Crackers from the street hawkers of Dhaka city as huge number of peoples also consume those non-branded items. Similarly, some non-branded bread items were also purchased from local confectionery shops. Table 1 represents the list of analyzed food items with brand and manufacturer names. Non-branded items are indicated as local made. In the case of potato chip samples, 20 samples from different manufacturers were collected. The same sampling pattern was followed for chanachur, bread, biscuits, cake, crisp-bread, cracker and breakfast cereal samples. French fries were collected from 20 different restaurants in Dhaka, Bangladesh.

**Table 1:** List of analyzed food items with brand and manufacturer names.

Name of the Food items	Brand name	Manufacturer Name
**1. Potato Chips**	Brand A	Manufacturer 1
	Brand B	Manufacturer 1
	Brand C	Manufacturer 2
	Brand D	Manufacturer 2
	Brand E	Manufacturer 2
	Brand F	Manufacturer 3
	Brand G	Manufacturer 4
	Brand H	Manufacturer 4
	Brand I	Manufacturer 4
	Brand J	Manufacturer 5
	Brand K	Manufacturer 6
	Brand L	Manufacturer 7
	Brand M	Manufacturer 7
	Brand N	Manufacturer 8
	Brand O	Manufacturer 9
	Brand P	Manufacturer 10
	Brand Q	Manufacturer 11
	Brand R	Manufacturer 12
	Brand S	Manufacturer 13
	Brand T	Manufacturer 14
**2. Chanachur**	Brand A	Manufacturer 1
	Brand B	Manufacturer 1
	Brand C	Manufacturer 2
	Brand D	Manufacturer 3
	Brand E	Manufacturer 3
	Brand F	Manufacturer 3
	Brand G	Manufacturer 3
	Brand H	Manufacturer 4
	Brand I	Manufacturer 5
	Brand J	Manufacturer 5
	Brand K	Manufacturer 5
	Brand L	Manufacturer 6
	Brand M	Manufacturer 7
	Brand N	Manufacturer 8
	Brand O	Manufacturer 8
	Brand P	Manufacturer 9
	Brand Q	Manufacturer 10
	Brand R	Manufacturer 11
	Brand S	Manufacturer 12
	Brand T	Manufacturer 13
**3. Crackers**	Brand A	Manufacturer 1
	Brand B	Manufacturer 1
	Brand C	Manufacturer 2
	Brand D	Manufacturer 2
	Brand E	Manufacturer 3
	Brand F	Manufacturer 4
	Brand G	Manufacturer 5
	Brand H	Manufacturer 6
	Brand I	Manufacturer 2
	Brand J	Manufacturer 7
	Brand K	Manufacturer 5
	Brand L	Manufacturer 5
	Brand M	Manufacturer 5
	Brand N	Manufacturer 8
	Brand O	Manufacturer 8
	Brand P	Manufacturer 7
	Brand Q	Manufacturer 9
	Brand R	Manufacturer 10
	Brand S	Manufacturer 11
	Brand T	Manufacturer 12
**4. Biscuits**	Brand A	Manufacturer 1
	Brand B	Manufacturer 2
	Brand C	Manufacturer 3
	Brand D	Manufacturer 4
	Brand E	Manufacturer 5
	Brand F	Manufacturer 4
	Brand G	
	Brand H	Manufacturer 6
	Brand I	Manufacturer 7
	Brand J	Manufacturer 8
	Brand K	Manufacturer 8
	Brand L	Manufacturer 9
	Brand M	Manufacturer 10
	Brand N	Manufacturer 1
	Brand O	Manufacturer 11
	Brand P	Manufacturer 12
	Brand Q	Manufacturer 13
	Brand R	Manufacturer 4
	Brand S	Manufacturer 14
	Brand T	Manufacturer 15
**5. Potato Crisps**	Brand A	Manufacturer 1
	Brand B	Manufacturer 2
	Brand C	Manufacturer 3
	Brand D	Manufacturer 4
	Brand E	Manufacturer 4
	Brand F	Manufacturer 5
	Brand G	Manufacturer 5
	Brand H	Manufacturer 5
	Brand I	Manufacturer 5
	Brand J	Manufacturer 5
	Brand K	Manufacturer 6
	Brand L	Manufacturer 7
	Brand M	Manufacturer 3
	Brand N	Manufacturer 8
	Brand O	Manufacturer 9
	Brand P	Manufacturer 10
	Brand Q	Manufacturer 11
	Brand R	Manufacturer 12
	Brand S	Manufacturer 13
	Brand T	Manufacturer 14
**6. Breakfast Cereals**	Brand A	Manufacturer 1
	Brand B	Manufacturer 1
	Brand C	Manufacturer 1
	Brand D	Manufacturer 1
	Brand E	Manufacturer 2
	Brand F	Manufacturer 3
	Brand G	Manufacturer 4
	Brand H	Manufacturer 2
	Brand I	Manufacturer 5
	Brand J	Manufacturer 6
	Brand K	Manufacturer 5
	Brand L	Manufacturer 7
	Brand M	Manufacturer 2
	Brand N	Manufacturer 8
	Brand O	Manufacturer 8
	Brand P	Manufacturer 9
	Brand Q	Manufacturer 5
	Brand R	Manufacturer 5
	Brand S	Manufacturer 10
	Brand T	Manufacturer 1
**7. French Fries**	Brand A	Manufacturer 1
	Brand B	Manufacturer 2
	Brand C	Manufacturer 3
	Brand D	Manufacturer 4
	Brand E	Manufacturer 5
	Brand F	Manufacturer 6
	Brand G	Manufacturer 7
	Brand H	Manufacturer 8
	Brand I	Manufacturer 9
	Brand J	Manufacturer 10
	Brand K	Manufacturer 11
	Brand L	Manufacturer 12
	Brand M	Manufacturer 13
	Brand N	Manufacturer 14
	Brand O	Manufacturer 15
	Brand P	Manufacturer 16
	Brand Q	Manufacturer 17
	Brand R	Manufacturer 18
	Brand S	Manufacturer 19
	Brand T	Manufacturer 20
**8. Cake**	Brand A	Manufacturer 1
	Brand B	Manufacturer 1
	Brand C	Manufacturer 2
	Brand D	Manufacturer 2
	Brand E	Manufacturer 2
	Brand F	Manufacturer 3
	Brand G	Manufacturer 4
	Brand H	Manufacturer 5
	Brand I	Manufacturer 6
	Brand J	Manufacturer 7
	Brand K	Manufacturer 8
	Brand L	Manufacturer 4
	Brand M	Manufacturer 5
	Brand N	Manufacturer 6
	Brand O	Manufacturer 7
	Brand P	Manufacturer 9
	Brand Q	Manufacturer 10
	Brand R	Manufacturer 11
	Brand S	Manufacturer 11
	Brand T	Manufacturer 12
**9. Bread**	Brand A	Manufacturer 1
	Brand B	Manufacturer 2
	Brand C	Manufacturer 2
	Brand D	Manufacturer 2
	Brand E	Manufacturer 2
	Brand F	Manufacturer 1
	Brand G	Manufacturer 3
	Brand H	Manufacturer 4
	Brand I	Manufacturer 1
	Brand J	Manufacturer 5
	Brand K	Manufacturer 6
	Brand L	Manufacturer 7
	Brand M	Manufacturer 8
	Brand N	Manufacturer 9
	Brand O	Manufacturer 10
	Brand P	Manufacturer 11
	Brand Q	Manufacturer 12
	Brand R	Manufacturer 13
	Brand S	Manufacturer 14
	Brand T	Manufacturer 15

Total 180 samples (each item 20 samples) were collected for this analysis. After collection, little amount of each sample was homogenized through a grinder (WBL15GM75, Walton, Bangladesh) and stored in proper temperature and light condition until further experimental procedures. One representative sample of every brand of each category was used for GC-MS/MS.

The total procedures were performed at the Institute of Food Science and Technology (IFST), Bangladesh Council for Scientific and Industrial Research (BCSIR), Dhaka, Bangladesh between November 2020 and May 2021.

### 2.3 Extraction

Acrylamide was extracted from the food samples according to the described protocol [25]. In summary, after homogenization, sample (1.5 g) was placed into a 50 ml centrifuge tube. The, homogenized samples were mixed with 500 μl of acrylamide-d3 solution and kept for about 10 min. In the next step, extraction was done at 60 °C in an ultrasonic bath (XUB10; Grant Instruments, UK). Later, the mix was centrifuged at 12,000 rpm for 15 min and mixed with n-hexane in 1:3 ratio and shaken for defatting. Next step is the Bromination step for overnight, where the collected water phase was added with 1 ml of 0.1 M/L HBr + KBrO_3_ and 1.5 g of KBr and excess Bromine was removed using 0.1 M sodium hyposulfite (1 ml). In the next step, extraction (2 times) was done with 4 ml of ethyl acetate. In the final step, sodium sulfate was used to evaporate the organic layer from the extracts and dissolved with ethyl acetate [26, 27] for analysis.

### 2.4 Sample analysis through GC-MS

For method validation and quantification of acrylamide contents in heat-processed and carbohydrate-rich foods of Dhaka, Bangladesh, GC-MS was applied. This study was performed in a GC-MS (TRACE 1310, Thermo Fisher Scientific, USA) equipped with a Thermo Scientific™ Trace GOLD™ TG-WAX GC Column (0.25 mm × 0.25 μm X 0.30 m) using Helium as carrier gas with 1 ml/min flow rate at 240 °C injection port temperature and sample injection volume of 2 μl. The temperature profile during this analysis was in the range of 50 °C–260 °C, ion traps was m/z 152, product ions were m/z 135 and m/z 155 and the product ion was m/z 137 for acrylamide dibromo and derivatives. The ion source temperature and collision energy was 230 °C and 1 V respectively. Ion peaks at m/z 135 and m/z 137 ratio areas were considered for acrylamide content detection. Spectra were detected through mass spectrometry (TSQ DUO, Thermos Scientific, USA). The results were calculated based on the mean value of three injections of each sample.

### 2.5 Method quality control

Only the blank samples were running for checking the column performance. Through injecting standard solutions of five different concentrations (5 ppb–200 ppb), the standard calibration curve was produced. Average blank value method was applied to detect the limit of detection (LOD) and limit of quantification (LOQ). For LOD determination, the signal-to-noise ratio (3:1) and for LOQ determination 10 times the value of baseline noise in the chromatogram of the blank samples was considered.

### 2.6 Recovery performance evaluation

Method performance was evaluated through recovery evaluation. For this, samples were fortified with two concentrations of acrylamide: 5 μg/L and 10 μg/L. Then, the extraction and detection was done using the verified method. This test was repeated at least two times and mean recovery (%) and standard deviation (SD) was calculated.

### 2.7 Method validation

**Figure 1:**
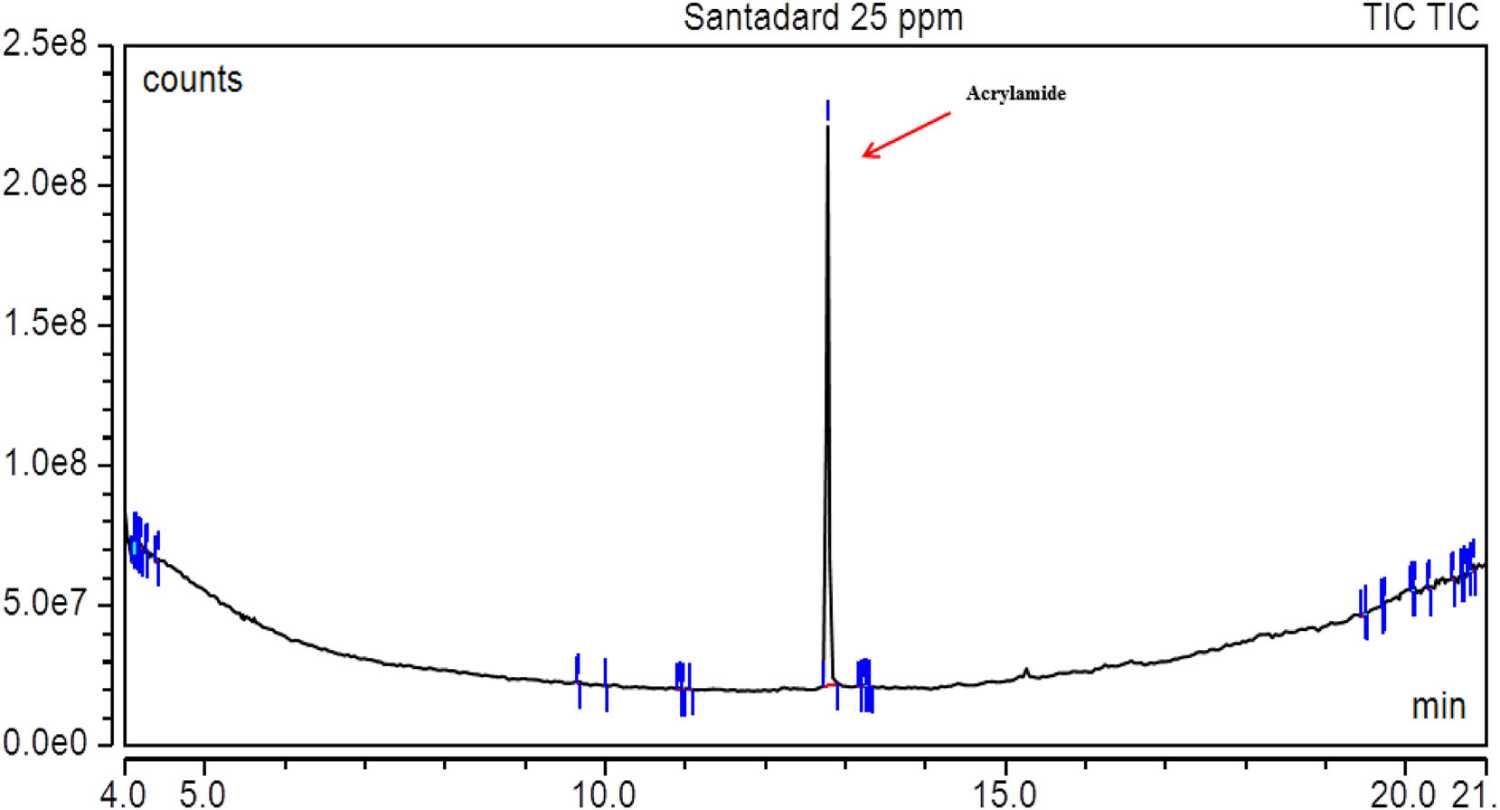
Chromatogram after injecting acrylamide 25 ppm standard solution into GC-MS/MS.

The coefficient of determination (R^2^) value was 0.989 detected from the standard curve produced from five different concentrations (5 ppb–200 ppb). The corresponding peak of acrylamide in standard solutions of two different concentrations is shown in Figure 1. The LOD (limit of detection) values were 11 μg/kg, 10 μg/kg, 8 μg/kg, 7 μg/kg, 11 μg/kg, 7 μg/kg, 12 μg/kg, 7 μg/kg and 6 μg/kg for potato chips, chanachur, potato crisps, biscuits, cake, bread, crackers, breakfast cereals and French fries, respectively. The LOQ (limit of quantification) values were 32 μg/kg, 33 μg/kg, 28 μg/kg, 30 μg/kg, 28 μg/kg, 29 μg/kg, 34 μg/kg, 27 μg/kg and 30 μg/kg for potato chips, chanachur, potato crisps, biscuits, cake, bread, crackers, breakfast cereals and French fries, respectively (Table 2). The differences in the acrylamide contents of the same products may be attributable to the use of raw materials from different sources, different heat treatment durations and sample pretreatment methods. After spiking with acrylamide, extraction and analysis of the spiked samples were performed using exactly the same procedures as the analyzed samples. The percent recoveries (5 μg/kg spiked) obtained in this study were 73 ± 4, 77 ± 3, 87 ± 5, 92 ± 4, 74 ± 3, 93 ± 5, 94 ± 5, 88 ± 3 and 82 ± 5 for potato chips, chanachur, potato crisps, biscuits, cake, bread, crackers, breakfast cereals and French fries, respectively, while the percent recoveries (10 μg/kg spiked) were 86 ± 4, 80 ± 5, 81 ± 5, 87 ± 5, 81 ± 5, 86 ± 3, 96 ± 6, 76 ± 5 and 80 ± 4 for potato chips, chanachur, potato crisps, biscuits, cake, bread, crackers, breakfast cereals and French fries, respectively (Table 3).

**Table 2:** **Acrylamide concentrations (μg/kg), SD, LOD and LOQ values of analyzed samples.** All of the products of the same category were purchased from different manufacturers. The manufacturing process, sample treatment and raw materials may differ from manufacturer to manufacturer.

Samples	Number of Samples	Mean	SD	Range	LOD (μg/kg)	LOQ (μg/kg)
Potato chips	20	730	293	153–1380	11	32
Chanachur	20	244	83	111–412	10	33
Potato Crips	20	598	222	278–1294	8	28
Biscuits	20	340	189	<LOQ-790	7	30
Cake	20	548	278	<LOQ-1054	11	28
Bread	20	217	77	<LOQ-370	7	29
Crackers	20	558	297	317–1129	12	34
Breakfast cereals	20	391	263	<LOQ-643	7	27
French fries	20	679	285	437–1227	6	30

SD: Standard Deviation; LOD: Limit of detection; LOQ: Limit of quantification.

**Table 3:** Obtained results from recovery test.

Samples	% recovery (5 μg/kg)	% recovery (10 μg/kg)
Potato chips	73 ± 4	86 ± 4
Chanachur	77 ± 3	80 ± 5
Potato Crips	87 ± 5	81 ± 5
Biscuits	92 ± 4	87 ± 5
Cake	74 ± 3	81 ± 5
Bread	93 ± 5	86 ± 3
Crackers	94 ± 5	96 ± 6
Breakfast cereals	88 ± 3	76 ± 5
French fries	82 ± 5	80. ± 4

## 3 Results and discussion

### 3.1 Determination of acrylamide

**Figure 2:**
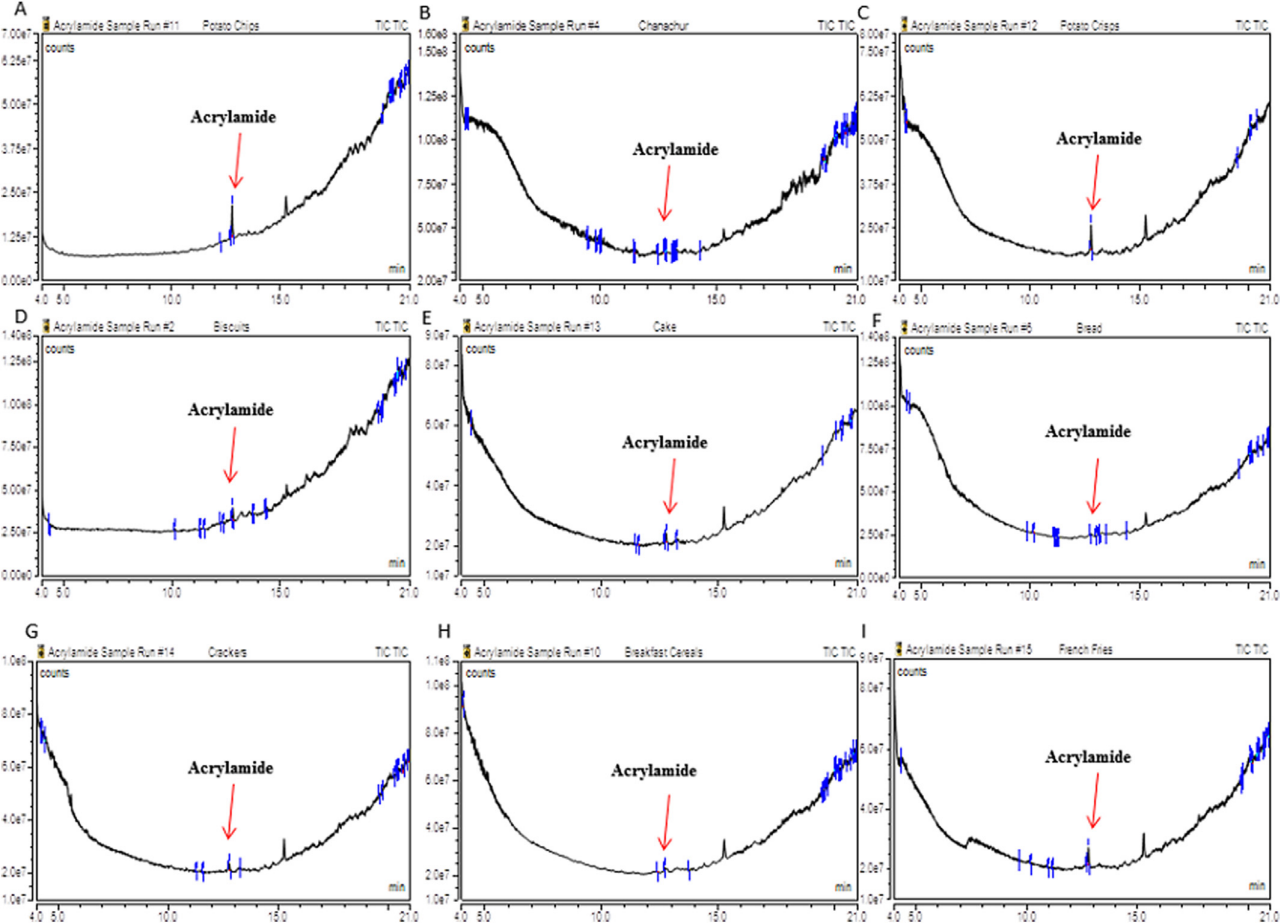
Chromatograms after injecting samples into GC-MS/MS. A: Potato chips; B: Chanachur; C: Potato Crisps; D: Biscuits; E: Cake; F: Bread; G: Crackers; H: Breakfast Cereals; I: French Fries.

From this analysis, detected acrylamide contents were 153–1380 μg/kg (mean value of 730 ± 293 μg/kg), 111–412 μg/kg (mean value: 244 ± 83 μg/kg), 278–1294 μg/kg (mean value: 598 ± 222 μg/kg), <LOQ-790 μg/kg (mean value: 340 ± 189 μg/kg), <LOQ-1054 μg/kg (mean value: 548 ± 278 μg/kg), <LOQ-370 μg/kg (mean value: 217 ± 77 μg/kg), 317–1129 μg/kg (mean value: 558 ± 297 μg/kg), <LOQ-643 μg/kg (mean value: 391 ± 263 μg/kg) and 437–1227 μg/kg (mean value: 679 ± 285 μg/kg) for potato chips, chanachur, crisis, biscuits, bread, cake, crackers, breakfast cereals and French fries, respectively (Table 2). The chromatographic peaks of acrylamide in samples is presented in Figure 2.

Based on this analysis, potato chips contained higher amount of acrylamide, followed by French fries, potato crisps, crackers, cake, breakfast cereals, biscuits, chanachur and bread samples. Normally, acrylamide is produced in high carbohydrate-rich foods that are processed through heat. Acrylamide is formed by reducing sugars and free asparagine [28, 29], and it was observed that acrylamide can be formed at temperatures lower than 100 °C [30]. The reducing sugar contents influence acrylamide formation in fried foods [31]. Blanching effects and reducing sugars influence acrylamide formation [32]. Several other possible factors, such as carbohydrate reactions, proteins, amino acid reactions and lipid reactions, are involved in acrylamide formation. In carbohydrate-rich foods, acrylamide is formed through a milliard reaction in which sugars and asparagine participate. Lipid oxidation can reduce acrylamide formation during frying and baking [33], while the same effect occurs after the addition of protein [34] after increasing the elimination reaction rate compared to the acrylamide formation reaction. Acrylamide is reactive in nature because, in food products, the monomeric form of acrylamide is formed after thermal treatment. The monomeric form is basically an amide with unsaturated double bonds that has polymerization capability when it dissolves or oxidizes into oxidative agents. The acrylamide level in specific food items may be determined through the acrylamide formation during food processing.

Variable acrylamide contents were detected in samples of the same category, which may be due to the ingredients used to produce those products, as different ingredients contained variable amounts of free asparagines and reducing sugars to form acrylamide. The acrylamide content was shown to decrease because of long-term heat treatment [35, 36, 37]. During acrylamide formation, several other reactions, such as decarboxylation and multistage elimination reactions occur. Excessive heat may reduce acrylamide formation because of the faster elevation of elimination reactions compared to the acrylamide formation process. Acrylamide formations can be reduced through proper storage and transportation of raw materials [38], adding amino acids, citric acid or hydrochloric acid. The antioxidants ascorbyl palmitate and sodium ascorbate reduce acrylamide contents by lowering pH and increasing water binding. After adding citric acid or hydrochloric acid, acrylamide formation decreased and was degraded [39]. In dry bread items and biscuits, the acrylamide contents can be reduced by lowering the temperature and increasing the baking time. The ingredients and formulations also influence acrylamide contents in foods. Sometimes the use of whole wheat flour and bran increases the acrylamide contents in biscuits, while biscuits produced from plain flour were found to contain low acrylamide contents [40]. The food items used in this study were of different brands from several manufacturers. The acrylamide concentrations in the same food items may be attributable to divergent food processing systems. The ingredients used during the manufacturing process might be from variable sources that have a great impact on acrylamide formation during processing.

For the production of potato chips, potato crisps, and French fries, raw materials (potato, oil) were collected from different parts of Bangladesh. In this case, the manufacturers collected their raw materials from different sources. Before collecting those materials for potato chips, potato crisps, and French fry production, the raw materials may be pretreated by suppliers, which may vary from supplier to supplier. Because of different pretreatments of raw materials by several different sources, acrylamide contents may vary among manufacturers. Similarly, differences in acrylamide contents in biscuits, cake, bread, cracks and breakfast cereals were observed because of different manufacturing procedures, processing times and pretreatments of raw materials by the suppliers. Among the analyzed food items, few differences in acrylamide contents were observed in chanachur samples. The differences may be because of the manufacturing process, raw material pretreatment and processing time.

Acrylamide contents were detected in carbohydrate-rich traditional Chinese foods at up to 771.1 μg/kg in China [41]. Acrylamide contents detected in most potato crisps were more than 1000 μg/kg in Japan [42], 998 μg/kg in Poland [25] and 968 ng/g in Italy [43]. Acrylamide content was detected in breads ranging from < LOQ to 695 μg/kg with a mean concentration of 225 μg/kg in Turkey [44]. Acrylamide concentrations in the crackers were detected in the range of 108–2180 μg/kg with a mean value of 630 μg/kg in Spain [45] and 604 μg/kg in Turkey [46]. In Turkey, biscuit samples were detected with a mean acrylamide concentration of 495 μg/kg [46]. Acrylamide concentrations were detected in biscuits, and breakfast cereals ranged from 30 μg/kg to 940 μg/kg in Italy [47]. In Bangladesh, acrylamide concentrations were detected in fried potato products, including potato chips, in the range of 197.04 μg/kg to 114.63 μg/kg, and lower acrylamide concentrations were detected in baked food items, with a range of 35.23–51.17 μg/kg [48]. In French fries, acrylamide contents were detected with a mean value of 401 μg/kg in Poland [49] and 303 μg/kg in Spain [50]. Similar trends of acrylamide occurrence in heat-treated carbohydrate-rich food products of Bangladesh were observed in the present study.

From this analysis, 15% of biscuits, 25% of bread, 20% of cake and 10% of breakfast cereal samples were detected with below the LOQ values. Analysis results indicated that 4 potato chip samples, 1 chanachur sample, 3 potato crisp samples, 3 biscuit samples, 2 cake samples, 3 bread samples, 4 crack samples, 2 breakfast cereal samples and 4 French fry samples out of 20 samples contained acrylamide levels above the benchmark levels set by the European Commission [EC] [51]. The nutritional facts and compositions of the analyzed food items are presented in Tables 4 and 5 respectively. The main ingredients of potato chips, potato crisps and French fries are potatoes. In this study, potato chips (approximately 63%) from some of the famous brands of Bangladesh were detected to have low acrylamide concentrations. The ingredients they used most frequently were potatoes, salt, different spices and flavors. The lowest acrylamide concentration was detected in one potato chip sample (153 μg/kg**),** where the overall protein content (7.43 g/100 g) was higher than that of the other samples. Otherwise, the thermal treatment and duration were almost the same for all of the chip samples of different brands. The highest acrylamide content was detected in potato chips of some local brands where the protein content was lower (6.98 g/100 g) and the thermal treatment was not applied for a long time. The potato crack samples used in this study also showed a similar trend to potato chips. The lowest acrylamide concentration was detected in the sample of one brand (278 μg/kg, where extra flavor was added), and the overall protein content was higher (7.32 g/100 g). In the case of French fry samples, the restaurants stored the raw materials before frying. The differences in the acrylamide concentrations might be because of the storage conditions of potatoes, as the lowest acrylamide concentration (437 μg/kg**)** was detected when using potatoes immediately without storage. The frying time might also have influenced the acrylamide concentration among the French fry samples. The lowest acrylamide contents were detected in chanachur samples because very few carbohydrates were used in almost all of the analyzed samples. The main ingredients of chanachur are wheat flour, lentils, peas and several spices. However, the lowest acrylamide content (111 μg/kg**)** was detected in one sample where the chanachur was very crispy, as it had been fried for a longer time and had high protein content (8.92 g/100 g). The main ingredients of biscuits, crackers, cakes, and breads are wheat flour, sugar and other supplementary items. In this study, the lowest acrylamide content (<LOQ) was detected in biscuit samples where either the heat treatment was not very high or the duration of heat treatment was shorter. In crack samples, variable acrylamide contents were detected in different brands because the raw materials were from different sources for every manufacturer. The manufacturing processes were similar for all of the manufacturers. Moreover, biscuits with lower acrylamide contents had higher protein contents (7.63 g/100 g), and extra flavor and emulsifiers were added. There may be an effect of the added flavor and emulsifiers on acrylamide formation during processing because the manufacturing process and all other compositions were almost the same. In the case of bread, breakfast cereals and cake samples, similar heat treatments were used in all of the brands of each sample category. The differences in the acrylamide contents among the samples of different brands might be attributable to the raw materials used, such as those from different sources, and might be stored in different storage conditions before processing.

**Table 4:** The Nutritional facts of all 20 brands of each sample category used in this study (per 100 g).

Food Items	Calorie (k. Cal)	Total Fat (g)	Cholesterol (mg)	Sodium (mg)	Total Carbohydrate (g)	Sugar (mg)	Protein (g)	Sodium Chloride (mg)
Potato chips	540–548	31–35	0	N/A	52–55	1–2	6.98–7.43	0.56–0.98
Chanachur	513–540	27.09–34.32	0	N/A	52–57	1–2	6.43–8.92	1900–2100
Potato Crisps	446–569	27.76–35.53	0	578–675	47–54	1.2–2.3	6.87–7.32	0.98–1.02
Biscuits	461–468	15.98–18.43	0	N/A	62.76–69.43	10.28–13.01	6.09–7.63	512–578
Cakes	412–478	17.76–21.83	0	0	52–54	22.65–28.41	5.97–6.5	0.41–0.58
Breads	378–397	3.09–8.54	0	30–90	78.98–88.02		7.45–8.54	N/A
Crackers	473–495	12.3–22.2	0	437–567	68.31–74.76	6.09–9.43	5.98–6.34	0.87–1.32
Breakfast Cereals	143–167		0	498–510	82.87–86.43	7.45–8.54	7.98–8.93	N/A
French Fries	512–547	27.21–31.59	0	354–378	45.87–55.32	8.05–14.32	6.02–6.26	112–190

**Table 5:** The food compositions of all 20 brands of each sample category used in this study.

Food Items	Compositions
Potato chips	Potato, Edible oil, sugar, salt and spices etc.
Chanachur	Chickpea, Flour, peanuts, Green beans, Lentils, Split chickpeas, Rice Flakes, Edible Oil, Spices, salt, Citric Acid, Red Chilli Powder, Powder and turmeric Powder etc.
Potato Crisps	Potato Flakes, Flour, Potato Starch, Vegetable oil, Salt, sugar, Baking Powder, seasoning powder etc.
Biscuits	Flour, Sugar, Edible Oil, Skim Milk powder, Salt, Emulsifiers, Anti-oxidants, Recommended feed flavors etc.
Cakes	Flour, Sugar, Egg, Water, Salt, Vegetable Oil, Fat, Glycerol, Starch, Skim Milk Powder, Raising agents, Potassium Sorbet, Emulsifiers, Sodium Acetate, Stabilizer, Recommended artificial Flavors etc.
Breads	Flour, Sugar, Vegetable oil, Salt, Yeast etc.
Crackers	Flakes, Flour, Potato Starch, Vegetable Oil, Salt, Sugar etc.
Breakfast Cereals	Processed Corn, Wheat, Oats and Barley etc.
French Fries	Potato, Spices, Salt, vegetable oil etc.

Bangladeshi people, especially young people, like heat-treated processed foods, and daily consumption rates are increasing in city areas [52]. Moreover, acrylamide formation varies among foods because of the storage conditions of raw materials, heat treatment during processing and different processing methods. The major implication of this study is that Bangladeshi people are aware of the acrylamide contents in regularly consumed carbohydrate-rich processed foods. However, because of the high consumption of heat-treated carbohydrate-rich processed foods, there are potential health risks for consumers of all ages. Therefore, proper protocols should be maintained during food preparation to reduce the acrylamide contents in food items.

## 4 Conclusions

The presence of acrylamide traces in potato chips, chanachur, potato crisps, biscuits, cake, bread, crackers, breakfast cereals and French fries collected from Dhaka, Bangladesh is reported in this study. The detected acrylamide contents in the range from below the LOQ to 1294 μg/kg. The obtained values showed almost similar trend with detected values of other countries. Acrylamide content varied among the samples of each group might be the effect of processing, raw materials and manufacturing process. Data from this study is useful for estimation of dietary intake of acrylamide for Bangladeshi population for risk assessment evaluation.

## Declarations

### Author contribution statement

G. M. M. Anwarul Hasan, Anuj Kumer Das: Conceived and designed the experiments; Performed the experiments; Analyzed and interpreted the data; Wrote the paper.

Mohammed A. Satter: Conceived and designed the experiments; Analyzed and interpreted the data; Contributed reagents, materials, analysis tools or data; Wrote the paper.

### Funding statement

This work was supported by the ADP project allocated by the Ministry of Science and Technology (MOST), People's Republic of Bangladesh.

### Data availability statement

Data included in article/supplementary material/referenced in article.

### Declaration of interests statement

The authors declare no conflict of interest.

### Additional information

No additional information is available for this paper.

## References

[1] Capuano E., Fogliano V. (2011). Acrylamide and 5-hydroxymethylfurfural (HMF): a review on metabolism, toxicity, occurrence in food and mitigation strategies. LWT-food science and technology.

[2] Hu Q., Xu X., Li Z., Zhang Y., Wang J., Fu Y., Li Y. (2014). Detection of acrylamide in potato chips using a fluorescent sensing method based on acrylamide polymerization-induced distance increase between quantum dots. Biosens. Bioelectron..

[3] Mottram D.S., Wedzicha B.L., Dodson A.T. (2002). Acrylamide is formed in the Maillard reaction. Nature.

[4] Stadler R.H., Blank I., Varga N., Robert F., Hau J., Guy P.A., Robert M.-C., Riediker S. (2002). Acrylamide from Maillard reaction products. Nature.

[5] Eriksson S. (2005).

[6] Ashoor S.H., Zent J.B. (1984). Maillard browning of common amino acids and sugars. J. Food Sci..

[7] Mellema M. (2003). Mechanism and reduction of fat uptake in deep-fat fried foods. Trends Food Sci. Technol..

[8] Mucci L.A., Wilson K.M. (2008). Acrylamide intake through diet and human cancer risk. J. Agric. Food Chem..

[9] Tareke E., Rydberg P., Karlsson P., Eriksson S., T¨ornqvist M. (2002). Analysis of acrylamide, a carcinogen formed in heated foodstuffs. J. Agric. Food Chem..

[10] Acrylamide I.A.R.C. (1994).

[11] Harris R.S., Karmas E., Harris R.S. (2014). Nutritional Evaluation of Food Processing.

[12] UK Food Standards Agency (2002). http://www.foodstandards.gov.uk/.

[13] Becalski A., Lau B.P.-Y., Lewis D., Seaman S.W. (2003). Acrylamide in foods: occurrence, sources, and modeling. J. Agric. Food Chem..

[14] Weisshaar R., Gutsche B. (2002). Formation of acrylamide in heated potato products – model experiments pointing to asparagine as precursor. Deut. Lebensm. Rundsch..

[15] Zyzak D.V., Sanders R.A., Stojanovic M., Tallmadge D.H., Eberhart B.L., Ewald D.K., Gruber D.C., Morsch T.R., Strothers M.A., Rizzi G.P., Villagran M.D. (2003). Acrylamide formation mechanism in heated foods. J. Agric. Food Chem..

[16] Clark J.P. (2003). Happy birthday, potato chip! and other snack developments. Food Technol..

[17] Baumann B., Escher E. (1995). Mass and heat transfer during deep fat frying of potato slices. I. Rate of drying and oil uptake. Lebensmittel-Wissenschaft und-Technologie.

[18] Garayo J., Moreira R. (2002). Vacuum frying of potato chips. J. Food Eng..

[19] Mellema M. (2003). Mechanism and reduction of fat uptake in deep-fat fried foods. Trends Food Sci. Technol..

[20] Yaylayan V.A., Wnorowski A., Perez L.C. (2003). Why asparagine needs carbohydrates to generate acrylamide. J. Agric. Food Chem..

[21] Xu Y., Cui B., Ran R., Liu Y., Chen H., Kai G., Shi J. (2014). Risk assessment, formation, and mitigation of dietary acrylamide: current status and future prospects. Food Chem. Toxicol..

[22] Hu Q., Xu X., Li Z., Zhang Y., Wang J., Fu Y., Li Y. (2014). Detection of acrylamide in potato chips using a fluorescent sensing method based on acrylamide polymerization-induced distance increase between quantum dots. Biosens. Bioelectron..

[23] (2005). Joint FAO/WHO Experts Committee on Food Additives (JECFA), Summary and Conclusions of the Sixty-Fourth Meeting of the Joint FAO/WHO Experts Committee on Food Additives (JECFA), Rome, 8–17 February.

[24] Roy T.S., Chakraborty R., Parvez M.N., Biswas S., Chakraborty S. (2017). Development of sustainable gross national income from potato export in Bangladesh-A perspective review. Univ. J. Agric. Res..

[25] Mojska H., Gielecnska I., Szponar L. (2007). Acrylamide content in heat-treated carbohydrate-rich foods in Poland. Rocz. Panstw. Zakl. Hig..

[26] Castle L. (1993). Determination of acrylamide monomer in mushrooms grown on polyacrylamide gel. J. Agric. Food Chem..

[27] Takere E., Rydberg P., Karlsson P., Eriksson S., Tornqvist M. (2002). Analysis of acrylamide, a carcinogen formed in heated foodstuffs. J. Agric. Food Chem..

[28] Biedermann M., Grob K. (2003). Model studies on acrylamide formation in potato, wheat flour and corn starch; ways to reduce acrylamide contents in bakery ware. Mittl. aus Leb. Hyg..

[29] Weisshaar R. (2004). Acrylamidein heated potato products– analytics and formation routes. Eur. J. Lipid Sci. Technol..

[30] Gutsche B., Weißhaar R., Buhlert J. (2002). Acrylamid in Lebensmitteln – ergebnisse aus der amtlichen Lebensmittelüberwachung Baden-Würtembergs. Dtsch. Lebensm.-Rundsch..

[31] Biedermann M., Grob K. (2003). Model studies on acrylamide formation in potato, wheat flour and corn starch; ways to reduce acrylamide contents in bakery ware. Mittl. aus Leb. Hyg..

[32] Haase N.O., Matthäus B., Vosmann K. (2004). Aspects of acrylamide formation in potato crisps. J. Appl. Bot. Food Qual..

[33] Capuano E., Oliviero T., Açar Ö.Ç., Gökmen V., Fogliano V. (2010). Lipid oxidation promotes acrylamide formation in fat-rich model systems. Food Res. Int..

[34] Claeys W.L., De Vleeschouwer K., Hendrickx M.E. (2005). Effect of amino acids on acrylamide formation and elimination kinetics. Biotechnol. Prog..

[35] Gutsche B., Weißhaar R., Buhlert J. (2002). Acrylamid in Lebensmitteln – ergebnisse aus der amtlichen Lebensmittelüberwachung Baden-Würtembergs. Dtsch. Lebensm.-Rundsch..

[36] Biedermann M., Grob K. (2003). Model studies on acrylamide formation in potato, wheat flour and corn starch; ways to reduce acrylamide contents in bakery ware. Mittl. aus Leb. Hyg..

[37] Weisshaar R. (2004). Acrylamidein heated potato products– analytics and formation routes. Eur. J. Lipid Sci. Technol..

[38] Eriksson S. (2005).

[39] Studer A., Blank I., Stadler R.H. (2004). Thermal processing contaminants in foodstuffs and potential strategies of control. Czech J. Food Sci..

[40] Taeymans D., Wood J., Ashby P., Blank I., Studer A., Stadler R.H., Gondé P., Eijck P., Lalljie S.A.M., Lingnert H., Lindblom M. (2004). A review of acrylamide: an industry perspective on research, analysis, formation, and control. Crit. Rev. Food Sci. Nutr..

[41] Zhang Y., Ren Y., Zhao H., Zhang Y. (2007). Determination of acrylamide in Chinese traditional carbohydrate-rich foods using gas chromatography with micro-electron capture detector and isotope dilution liquid chromatography combined with electrospray ionization tandem mass spectrometry. Anal. Chim. Acta.

[42] Ono H., Chuda Y., Ohnishi-Kameyama M., Yada H., Ishizaka M., Kobayashi H., Yoshida M. (2003). Analysis of acrylamide by LC-MS/MS and GC-MS in processed Japanese foods. Food Addit. Contam..

[43] Esposito F., Nardone A., Fasano E., Triassi M., Cirillo T. (2017). Determination of acrylamide levels in potato crisps and other snacks and exposure risk assessment through a Margin of Exposure approach. Food Chem. Toxicol..

[44] Boyacı Gündüz C.P., Cengiz M.F. (2015). Acrylamide contents of commonly consumed bread types in Turkey. Int. J. Food Prop..

[45] Mesías M., Morales F.J. (2015). Acrylamide in commercial potato crisps from Spanish market: trends from 2004 to 2014 and assessment of the dietary exposure. Food Chem. Toxicol..

[46] Gündüz C.P.B., Bilgin A.K., Cengiz M.F. (2017). Acrylamide contents of some commercial crackers, biscuits and baby biscuits. Akademik Gida.

[47] Capei R., Pettini L., Nostro A.L., Pesavento G. (2015). Occurrence of acrylamide in breakfast cereals and biscuits available in Italy. J. Prevent. Med. Hyg..

[48] Khan M., Moniruzzaman M., Razu M.H. (2019). Method Development and validation for the quantification of acrylamide in potato chips and other locally available food by LC-MS/MS in Bangladesh. Food Nutr. Sci..

[49] Mojska H., Gielecińska I., Małecka K. (2010). Determination of acrylamide content in potato products using GC-MS/MS and LC-MS/MS methods. Rocz. Panstw. Zakl. Hig..

[50] Mesías M., Morales F.J. (2015). Acrylamide in commercial potato crisps from Spanish market: trends from 2004 to 2014 and assessment of the dietary exposure. Food Chem. Toxicol..

[51] European Commission [EC] (2013).

[52] Banik R., Naher S., Pervez S., Hossain M.M. (2020). Fast food consumption and obesity among urban college going adolescents in Bangladesh: a cross-sectional study. Obes. Med..

